# A One-Armed Phase I Dose Escalation Trial Design: Personalized Vaccination with IKKβ-Matured, RNA-Loaded Dendritic Cells for Metastatic Uveal Melanoma

**DOI:** 10.3389/fimmu.2022.785231

**Published:** 2022-02-04

**Authors:** Elias A. T. Koch, Niels Schaft, Mirko Kummer, Carola Berking, Gerold Schuler, Kenichiro Hasumi, Jan Dörrie, Beatrice Schuler-Thurner

**Affiliations:** ^1^Department of Dermatology, Universitätsklinikum Erlangen, Friedrich-Alexander-University Erlangen-Nürnberg (FAU), Erlangen, Germany; ^2^Comprehensive Cancer Center Erlangen-European Metropolitan Region of Nuremberg (CCC ER-EMN), Erlangen, Germany; ^3^Deutsches Zentrum Immuntherapie (DZI), Erlangen, Germany; ^4^ICVS Tokyo Clinic, Chiyoda-ku, Japan

**Keywords:** metastatic uveal melanoma, tumor antigen vaccine, personalized vaccine, Immune checkpoint blockade, tumor antigens, IKKβ-matured dendritic cells

## Abstract

Uveal melanoma (UM) is an orphan disease with a mortality of 80% within one year upon the development of metastatic disease. UM does hardly respond to chemotherapy and kinase inhibitors and is largely resistant to checkpoint inhibition. Hence, further therapy approaches are urgently needed. To improve clinical outcome, we designed a trial employing the 3^rd^ generation personalized IKKβ-matured RNA-transfected dendritic cell (DC) vaccine which primes T cells and in addition activates NK cells. This ongoing phase I trial [NCT04335890 (www.clinicaltrials.gov), Eudract: 2018-004390-28 (www.clinicaltrialsregister.eu)] investigates patients with treatment-naive metastatic UM. Monocytes are isolated by leukapheresis, differentiated to immature DCs, matured with a cytokine cocktail, and activated *via* the NF-κB pathway by electroporation with RNA encoding a constitutively active mutant of IKKβ. Three types of antigen-RNA are co-electroporated: i) amplified mRNA of the tumor representing the whole transcriptome, ii) RNA encoding driver mutations identified by exome sequencing, and iii) overexpressed non-mutated tumor antigens detected by transcriptome sequencing. This highly personalized DC vaccine is applied by 9 intravenous infusions in a staggered schedule over one year. Parallel to the vaccination, standard therapy, usually an immune checkpoint blockade (ICB) as mono (anti-PD-1) or combined (anti-CTLA4 and anti-PD-1) regimen is initiated. The coordinated vaccine-induced immune response encompassing tumor-specific T cells and innate NK cells should synergize with ICB, perhaps resulting in measurable clinical responses in this resistant tumor entity. Primary outcome measures of this trial are safety, tolerability and toxicity; secondary outcome measures comprise overall survival and induction of antigen-specific T cells.

## Introduction

Uveal melanoma (UM) is the most common tumor of the eye in adults but still represents a rare subtype of melanoma. The mean age-adjusted incidence is 5.1 per million in the United States, and shows a north to south gradient in Europe from a minimum of 1.5 per million in southern Italy up to 9.2 per million in Ireland (1–3). The primary tumor of UM can be treated successfully regarding clearance and low intraocular recurrence rates. The treatment approach depends on the tumor size, patient preference, and tumor localization, most commonly including brachytherapy or enucleation (4, 5). Nevertheless, at least 40-50% of patients will develop metastases depending on genetic aberrations of the tumor, which spread predominantly to the liver (6, 7). Since there is no effective standard treatment for advanced UM, the prognosis is bleak once metastasis develops (8). A meta-analysis of a population including 912 patients from clinical trials showed a median overall survival (OS) of 10.2 months, whereby 15% received checkpoint blockade (ICB) and 34% chemotherapy (9). Recently published phase II trials of patients treated with combined ICB showed a more favorable OS of 19.1 and 12.7 months (10, 11). These data are in line with results in a real-world setting and are better than the proposed values before the ICB era (12–15). However, the therapeutic benefit of ICB is small, and at the cost of severe immune-related adverse events (AE) potentially involving all organ systems by a broad activation of the immune system. These AE can be challenging to manage, often require treatment interruption, systemic immunosuppression, and, in case of intolerable toxicity, a treatment discontinuation (16–18). Grade 3–4 drug-related AE occur in up to 57% of patients treated with nivolumab plus ipilimumab (10, 11, 14). Tebentafusp (IMCgp100), a first-in-class soluble T-cell engager (so-called ImmTACs), achieved for the first time prolongation of OS in a randomized trial in metastatic uveal melanoma, and is currently awaiting approval in the United States. The drug consists of a soluble TCR, binding to gp100 peptide/HLA-A*02:01 complexes, which is fused to an anti-CD3 scFv. Following signs of clinical efficacy in early clinical trials (NCT02570308) (19), a recent phase III trial demonstrated a clinically meaningful, significant improvement of OS as first-line treatment (median OS 21.7 versus 16.0 months for patients randomized to investigator´s choice, i.e., pembrolizumab, ipilimumab or dacarbazine; P<0.0001; stratified HR 0.51 [95%CI 0.37-0.71]) (20). Progression-free survival was also improved compared to ICB (3.3 versus 2.9 months, P=0.0139; HR 0.73). Interestingly, the ORR remained low (9% with IMCgp100 versus 5% with ICB) yet prolonged survival could be observed even in patients who progressed. This novel therapy seems relatively safe with cutaneous (rash, pruritus) and cytokine-mediated side effects (pyrexia, cytokine-release syndrome) but no treatment-related death and a low discontinuation rate (2% versus 4.5% with IC). Despite this advance, the prognosis for metastatic uveal melanoma stays dismal, notably for HLA-A*02:01 negative patients who do not qualify for IMCgp100 therapy. Thus, further therapeutic approaches for advanced UM are urgently needed. Building on our expertise with RNA-transfected 2^nd^ generation dendritic cell (DC) vaccines (NCT00074230, NCT01983748), and promising observations in compassionate treatments of UM (21), we designed a trial employing the 3^rd^ generation personalized IKKβ-activated RNA-transfected DC vaccine (22). By activation of NF-κB *via* the introduction of a constitutively active stabilized mutant of IKKβ, these DCs express increased levels of co-stimulatory molecules, induce better memory CTL responses, and are in addition effective activators of NK cells (23, 24). The DCs are loaded with amplified tumor mRNA and an individualized selection of shared UM-associated antigens and driver mutations based on exome and transcriptome sequencing of a tumor biopsy.

## Methods and Analysis

We are currently performing the phase I trial (NCT04335890) in patients with recently diagnosed metastatic UM, which is not curable with local therapy. We intend to treat and fully evaluate 12 patients aged between 18 and 75 years and irrespective of race and gender with a WHO performance status of 0, 1, or 2. Patients must have accessible metastases, which can be biopsied or surgically removed to obtain material for total tumor mRNA production and next generation sequencing (NGS). After tumor sampling, we extract RNA and DNA. mRNA is subjected to whole transcriptome sequencing and DNA to whole exome sequencing. With the help of the exome data, we select highly expressed antigens from a predesigned warehouse of UM antigen-encoding mRNAs: Tyrosinase, gp100, PRAME, MAGE-A3, and IDO1. For IDO1 a functionally inactive mutant was designed to circumvent its immunosuppressive activity. The exome data are also used to determine which common driver mutations are present in each individual tumor. The corresponding mRNAs are taken from a predesigned warehouse: GNAQ_R183Q_, GNAQ_Q209L/P_, GNA11_Q209L/P_, SF3B1_R625H/C_, CYSLTR2_L129Q_, and PLCB4_N630Y_. The use of a prefabricated warehouse of common antigens and driver mutations allows the production of a highly individualized vaccine in a very short time. To facilitate both MHC class I and II-restricted presentation, the antigens are equipped with the leader peptide from Lamp-1 (amino acid 1-27; GenBank: AAL58070.1) and the 40 C-terminal amino-acids from Lamp-3 (DC-LAMP; GenBank: AAH32940.1) which direct them to both presentation pathways (25).

**Figure 1:**
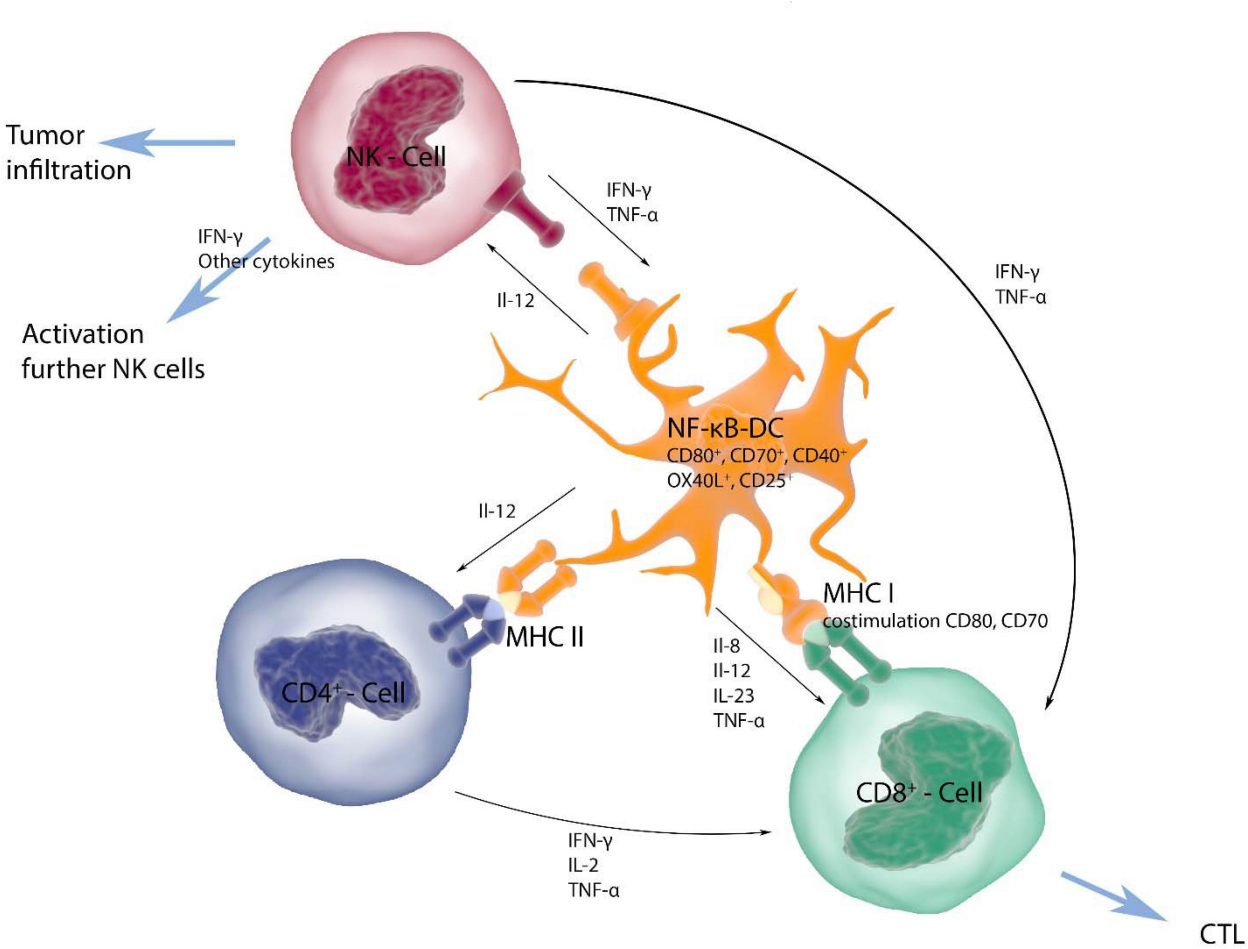
DCs present tumor antigens through MHC class I and II to CD8^+^ and CD4^+^ T cells, respectively, and are able to activate innate immune cells, like NK cells. T-cell priming is supported by co-stimulatory molecules and the secretion of pro-inflammatory cytokines. NF-κB-activated DCs express increased levels of the classical co-stimulatory molecules CD80 and CD86, but also of CD70, CD40, OX40L, and several pro-inflammatory cytokines, such as IL-12 and TNFα, which support induction of memory T-cell responses and increase NK-cell activation and proliferation. The antigens used in this trial are modified in such a way, that both MHC class I- and class II-restricted presentation is facilitated. Therefore, a comprehensive cellular response of helper T cells, cytotoxic T cells, and NK cells is induced, which in turn produce additional cytokines that support activation.

To obtain the required numbers of autologous DCs, a monocyte concentrate is collected *via* leukapheresis. Monocytes are purified by counterflow-elutriation and subsequently differentiated to immature DCs over 6 days in RPMI1640 supplemented with 1% autologous plasma, l-glutamine, GM-CSF, and IL-4. Then cells are exposed to a standard maturation cytokine cocktail (TNFα, IL-1β, IL-6, and PGE_2_) (26) for 24 h. After the DCs are matured, they are divided into three equal parts. One batch is electroporated with the respective common uveal melanoma tumor-associated antigens (gp100, tyrosinase, PRAME, MAGE-A3, IDO1), one batch with RNAs coding for common driver mutations known to occur in uveal melanoma (GNAQ, GNA11, SF3B1, CYSLTR2, PCLB4) and one batch with autologous tumor RNA (**Table 1**). No more than 3 antigens will be used in one batch. In addition, all DCs are electroporated with mRNA coding for a constitutively active, stabilized variant of IKKβ to activate the NF-κB pathway. Electroporation conditions are: square wave pulse, 1250 V/cm, pulse time 1 ms, described in detail elsewhere (27). These super-activated DCs (NF-κB-DCs) produce inflammatory cytokines, induce better memory CTL responses, and also activate NK cells (**Figure 1**) (24).

**Table 1:** Description of the antigen platform.

	Targets	Description
Tumor-associated antigens	Glycoprotein 100 (gp-100)	Transmembrane glycoprotein, highly expressed in normal melanocytes and melanoma cells.
	PRAME	PRAME is an antigen that is predominantly expressed in melanomas with a low expression in non-tumor tissue.
	Indoleamine-pyrrole 2,3-dioxygenase (IDO)	An enzyme expressed by tumor cells in response to inflammation and limits T-cell function. Plays a potential role in the immune escape mechanism.
	Melanoma-associated antigen 3 (MAGE-A3)	MAGE-A3 is a tumor-specific protein. It has been identified on several tumor entities including uveal melanoma. The function is unknown.
	Tyrosinase	A copper-containing enzyme in the melanosomes, which plays an important role in the melanogenesis.
Driver mutations	GNAQ	Driver mutation leads to activation of G protein-coupled receptor (GPCR) and to the carcinogenesis with no correlation to OS.
	GNA11	Driver mutation leads to activation of G protein-coupled receptor (GPCR) and to the carcinogenesis with no correlation to OS.
	SF3B1	Hot-spot mutation correlating with a peak of metastases after 7 years (intermediate risk of metastases).
	Cysteinyl leukotriene receptor 2 (CYSLTR2)	CYCLTR2 mutation activates Gαq in tumors lacking GNAQ, GNA11, and PLCB4 mutations promoting tumorigenesis without initiating metastasis.
	Phospholipase C β4 (PCLB4)	Mutation in the PCLB4 activates signaling downstream of GPCR by directly binding Gαq promoting tumorigenesis without initiating metastasis.
Autologous tumor RNA		Autologous RNA is extracted from a patient’s tumor sample obtained at biopsy or surgery. Total tumor RNA is extracted from the tumor cells, and tumor RNA is amplified using PCR amplification of a complementary DNA (cDNA) intermediate followed by *in vitro* transcription.

NF-κB-DCs get divided in three parts and electroporated with either (I) common uveal melanoma tumor-associated antigens, (II) RNAs coding for common driver mutations known to occur in uveal melanoma, or (III) autologous tumor RNA.

**Figure 2:**
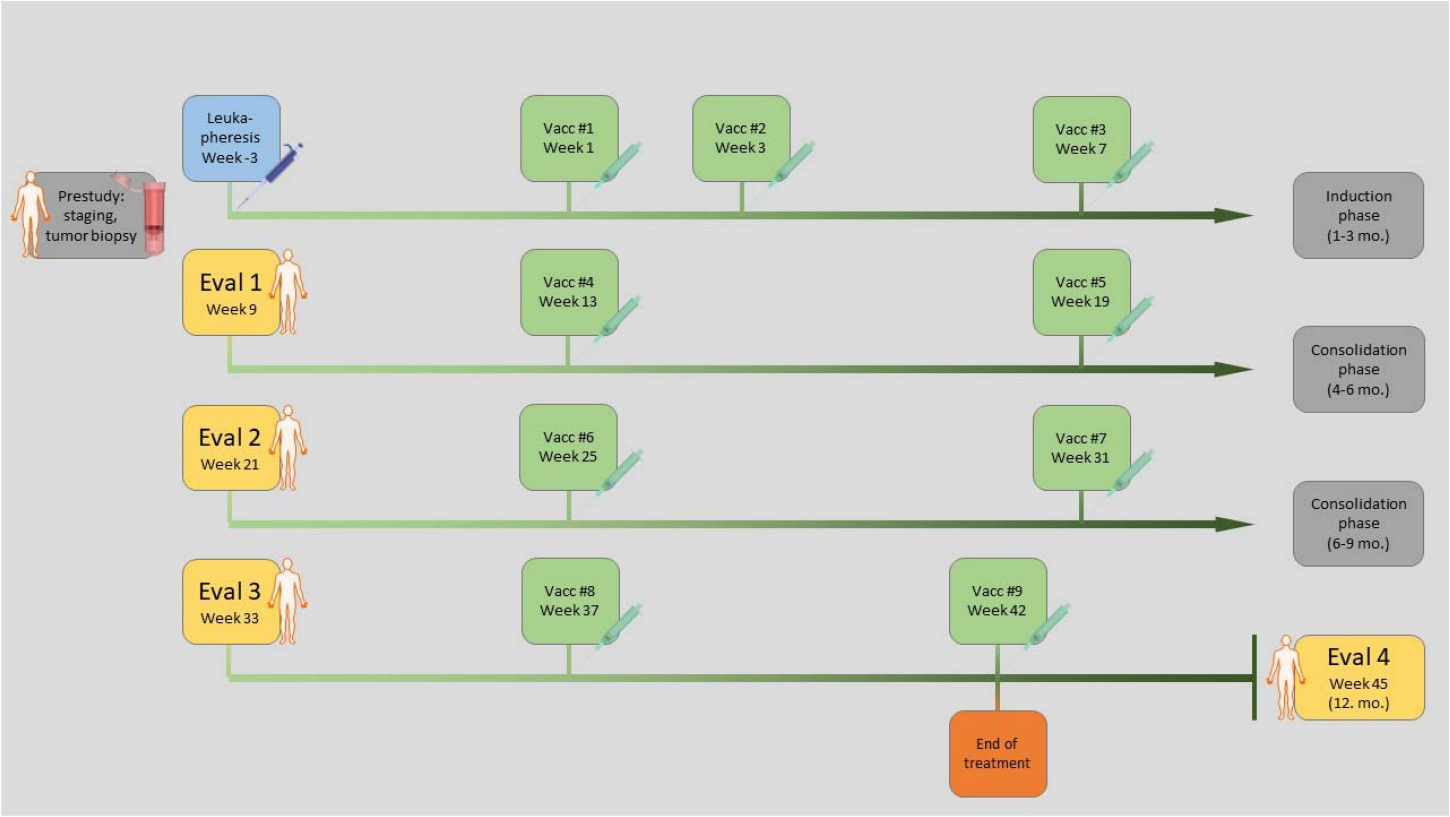
Treatment schedule: Initially a tumor biopsy is taken, and RNA and DNA preparation and sequencing is performed. To obtain the required numbers of autologous DCs, a monocyte concentrate is collected *via* leukapheresis. The vaccine is generated by RNA-electroporation of DCs and 9 vaccine injections are given intravenously over a period of one year. Every three months CT/MRI staging is performed.

Four hours after the electroporation process, the DC are mixed, cryopreserved and stored in cryo vials (consisting of 15 x 10^6^ cells, autologous serum, 10% DMSO, and 5% glucose) in the gas phase of liquid nitrogen (< -150°C) under appropriate, continuous monitoring of temperature. The NF-κB-DCs are tested for sterility and can only be administered to the patient afterwards. We expect 50 to 80% of the DCs to survive the procedure of both electroporation and cryoconservation, as we described earlier (28). At the visits, the DCs are thawed, resuspended in 50 ml 0,9% NaCl and administered intravenously to the patient within half an hour. The vaccine is applied with an intra-patient dose escalation of 7.5 to 30 million cells separated into three cohorts with increasing starting dose (**Table 2**). The vaccines are scheduled for 9 visits in increasing intervals of 2, 4, and 6 weeks over one year. In parallel to the vaccination, the patients receive the standard treatment for metastatic UM guided by a multi-disciplinary tumor board decision, preferably ICB as either mono- (PD-1 antibodies) or combined (PD-1 and CTLA-4 antibodies) treatment. The two therapies are administered independently in their appropriate interval with a minimum distance of 2 days to each other. Every three months, a tumor staging including MRI- and CT-scans is performed (**Figure 2**).

**Table 2:** Vaccination schedule with 9 visits.

Patient number	Vacc #1	Vacc #2	Vacc #3	Vacc #4	Vacc #5	Vacc #6	Vacc #7	Vacc #8	Vacc #9
**1-4**	7.5 x10^6^	7.5 x10^6^	15 x10^6^	15 x10^6^	30 x10^6^	30 x10^6^	30 x106	30 x10 ^6^	30 x10^6^
**5-8**	15 x10^6^	15 x10^6^	30 x10^6^	30 x10^6^	30 x10^6^	30 x10^6^	30 x10^6^	30 x10^6^	30 x10^6^
**8-12**	30 x10^6^	30 x10^6^	30 x10^6^	30 x10^6^	30 x10^6^	30 x10^6^	30 x10^6^	30 x10 ^6^	30 x10^6^

Patients will be vaccinated in a staggered approach by selectively decelerating release of the vaccine in increasing intervals of 2, 4, and 6 weeks.

Primary outcome measures within a period of one year are safety, dose-limiting toxicities, and maximum tolerated dose assessed by side effects using the Common Toxicity Criteria (CTC v4.0), as well as tolerability assessed by quality of life questionnaire (EORTC QLQ-C30, Version 2). Secondary pre-defined outcome measures are the 2-year OS and the induction of antigen-specific CD8^+^ T cells and/or CD4^+^ T cells against tumor-associated antigens and mutated drivers.

Due to the explorative nature of this phase I trial, all analyses will be descriptive and no confirmatory statistical testing is intended. The sample size of 12 evaluable patients was chosen as this number is sufficient to detect effects that increase in frequency from 5% in the population to 30% in the treated individuals with a power of over 80% and false positive probability of under 5%. The same applies to effects with a 10% frequency in the population and a 40% frequency in the treated patients.

## Discussion

Vaccination with peptide-loaded, monocyte-derived and cytokine-matured DCs (1^st^ generation DCs) induced tumor-specific class I and class II responses linked to an increased median overall survival (29). Such DCs, yet loaded with antigen-coding mRNAs rather than peptides (2^nd^ generation DCs), in a subsequent phase I/II trial (NCT00074230) in 82 metastasized cutaneous melanoma patients, also induced the highest vaccine-specific immune responses in long-term survivors (> 6 years), again without significant side effects (30).

The use of the highly potent, IKKβ-activated, RNA-loaded designer DCs (3^rd^ generation DCs) as adjuvant, combined with an individual selection of tumor neo-antigens and overexpressed antigens in this first-in-human trial holds great promise. Based on preclinical data and the study design we expect that i) more and better tumor-specific helper as well as cytotoxic T cells are induced in the UM patients, ii) NK cells get also activated resulting in a coordinated adaptive and innate immune response, and iii) that these immune responses are supported by ICB. Therefore, we expect that a prolonged survival may be achieved compared to ICB alone. However, this chance comes with potential risks of toxicity for the treated individuals, but experience with another type of highly activated DCs suggests that these should be manageable (31).

Although expressing tumor drivers in cellular drug products may raise the concern of malignant transformation of these cells, our antigen constructs are highly unlikely to induce cancer, because they only consist of small parts of the functional proteins. In addition, the cell type we are using is terminally differentiated and no malignancies arising from these cells have been described so far. Most importantly, the introduced proteins are expressed in a transient fashion by mRNA electroporation, which makes a functional integration into the genome of any cell impossible.

Concerning the induction of autoimmunity induced against the somatically mutated driver epitopes, we consider this risk lower than for non-mutated tumor antigens that have been used for decades without inducing dangerous autoimmunity. The beauty of this new class of tumor epitopes is their specificity exclusively for the tumor because they are only mutated in the malignant tissue, while all non-malignant tissues do not harbor the mutation. Hence, we think these antigens are both more efficient against the tumor, and less prone of inducing any autoimmune response. Further, the use of highly expressed antigens from a predesigned warehouse of UM antigen-encoding mRNAs seems promising, as these antigens have been used in previous vaccination trials without causing severe autoimmunity. All antigens except for IDO were even used in trials with mRNA-electroporated DCs [reviewed in (32)]. IDO plays potentially a role in the immune escape mechanism of UM as it is upregulated as response to IFN-γ and it might be necessary as a therapy target for a sustainable immune response (33). However, IDO is ubiquitously expressed but also plays a role in immune-protection of human corneal cells and could lead to autoimmunity (34) Nevertheless, it was shown to be a safe target in a peptide vaccination trial (35). Despite combination with ipilimumab and detection of IDO-specific T cells, no autoimmunity was observed (35). MAGE-A3 and PRAME are rarely expressed in somatic tissue and harbor a low risk for autoimmunity. The antigens gp100 and tyrosinase are also expressed by melanocytes and can lead to cutaneous adverse events as one could see in the IMCgp100 trial with the occurrence of 83% rash, 69% pruritus, 25% erythema, 21% skin exfoliation, 20% hair color changes, and 16% vitiligo (36). Nevertheless, both are well established melanoma antigens for immunotherapy, which have been used in various clinical trials with DCs (32).

The special feature of the NF-κB-DCs we test in this trial is their ability to produce cytokines, most importantly biologically active IL-12. This facilitates the formation of a cellular immunological memory, but since cytokine release syndrome is a major risk in other cellular immunotherapies, this topic needs to be addressed: The maximum tolerated dose for IL-12 is above 100 ng/kg and the lowest dose observed to cause mild side effects is 3 ng/kg (37, 38). Our maximum vaccination dose of 30 million DCs would result in a dose of only 0.6 ng/kg in patients with 75 kg body weight, because one million of DCs produce around 1.66 ng (39). For other cytokines, produced by the NF-κB-activated DCs the relations of doses are similar. Hence, we think that the produced cytokines will be effective in the microenvironment during DC/T-cell interaction to induce a favorable T-cell phenotype, but will not have toxic systemic effects.

Measuring the T-cell responses induced by the vaccine will be challenging, since the use of full-length antigens results in all possible HLA-restricted peptides. To tackle this, we have established an EliSpot-based method, which uses again mRNA-electroporation for antigen-loading (40). This method is HLA-haplotype independent, and will give initial information, which patients responded to which antigen. Those will then be analyzed in more detail, using epitope prediction algorithms like netMHC (41) and multicolor-combicoded HLA-peptide-multimers (42). Alternatively, T cells can be stimulated by mRNA-transfected targets and then be subjected to a MANAFEST-assay (43), to determine specific reactivity. The immunological responses may then be correlated with the clinical outcome.

## Conclusions

We anticipate that our 3^rd^ generation DC vaccine induces high-quality tumor-specific helper and cytotoxic T cells and activates NK cells. The use of a prefabricated warehouse of antigens to select from based on individual NGS-data of the tumor allows the rapid production of a highly individualized cellular vaccine. The specific and coordinated immune response should synergize with the block of inhibitory checkpoints, perhaps resulting in measurable clinical responses in this resistant tumor entity.

The first results from this trial are expected in 2022.

## Data Availability Statement

The original contributions presented in the study are included in the article. Further inquiries can be directed to the corresponding author.

## Ethics Statement

The studies involving human subjects were reviewed and approved by Ethikkommission of the Friedrich-Alexander University Erlangen-Nürnberg. The patients provided their written informed consent to participate in this study. The principal investigator and all other investigators ensure that this study will be conducted in full conformity with the principles set forth in the Declaration of Helsinki. It will be carried out in accordance with Good Clinical Practice (GCP) as required by the “Verordnung über die Anwendung der Guten Kl inischen Praxis bei der Durchführung von klinischen Prüfungen mit Arzneimitteln zur Anwendung am Menschen (GCP-Verordnung) vom 9. August 2004”, based upon the guidance 2001/20/EG of the European Parliament and the ICH Guideline for Good Clinical Practice (CPMP/ICH/135/95) July 2002. Furthermore the trial is conducted according to the regulations described in the Arzneimittelgesetz AMG (e.g. §13-20 production, § 40 to 42 Clinical trials). The Experimentelle Immuntherapie, Hautklinik, Universitätsklinikum Erlangen which will be producing the dendritic cell vaccine has legal approval for GMP production of tumor-peptide-loaded as well as RNA-transfected autologous dendritic cells (Manufacturing License issued by the Regierung von Oberbayern on the 16th July 2009 according to AMG § 13 Absatz 1; certificate DE_BY 04_MIA_2009_0146/53.2 - ZAB - 2671.1 H 207). The trial is approved by the Paul Ehrlich Institute (PEI).

## Author Contributions

EK, JD, and BS-T wrote the draft of the manuscript. EK designed the figures. All authors are involved in the design, planning, or performance of the clinical trial. All authors have read and approved the final manuscript.

## Funding

This research was funded by Hasumi International Research Foundation, 2200 Pennsylvania Avenue, NW, 4th Floor East, Washington, DC 20037, USA. Tel: + 1 202 973-6459. E-Mail: office@hasumi-foundation.org.

## Conflict of Interest

The authors declare the following potential conflict of interest: GS, NS, and JD are named as inventors on a patent on caIKK-RNA-electroporated DCs (WO/2012/055551), which is held by the Friedrich-Alexander-Universtät Erlangen-Nürnberg.

The remaining authors declare that the research was conducted in the absence of any commercial or financial relationships that could be construed as a potential conflict of interest.

## Publisher’s Note

All claims expressed in this article are solely those of the authors and do not necessarily represent those of their affiliated organizations, or those of the publisher, the editors and the reviewers. Any product that may be evaluated in this article, or claim that may be made by its manufacturer, is not guaranteed or endorsed by the publisher.
